# Promoter methylation of SEPT9 as a potential biomarker for early detection of cervical cancer and its overexpression predicts radioresistance

**DOI:** 10.1186/s13148-019-0719-9

**Published:** 2019-08-19

**Authors:** Xinlin Jiao, Siying Zhang, Jun Jiao, Teng Zhang, Wenjie Qu, Guy Mutangala Muloye, Beihua Kong, Qing Zhang, Baoxia Cui

**Affiliations:** 1 Department of Obstetrics and Gynecology, Qilu Hospital, Shandong University, 107 West Wenhua Road, Ji’nan, 250012 Shandong People’s Republic of China; 2 Gynecology Oncology Key Laboratory, Qilu Hospital, Shandong University, Ji’nan, 250012 Shandong People’s Republic of China

**Keywords:** Cervical cancer, SEPT9, Methylation, HMGB1-RB axis, Radio-resistance, TAMs polarization

## Abstract

**Background:**

Cervical cancer screening by combined cytology and HPV test has reduced the incidence of cervical cancer, but cytological screening lacks a higher sensitivity while HPV testing possesses a lower specificity. Most patients with invasive cervical cancer are treated with radiotherapy. However, insensitivity to radiotherapy leads to poor efficacy.

**Methods:**

Illumina Methylation EPIC 850k Beadchip was used for genomic screening. We detected methylation of SEPT9 and mRNA expression in different cervical tissues by using methylation-specific PCR and qRT-PCR. Then using CCK8, migration assay, and flow cytometry to detect the biological function and irradiation resistance of SEPT9 in vitro and in vivo. Liquid chromatography coupled with tandem mass spectrometry (LC-MS/MS) and co-immunoprecipitation (CoIP) were used to find the interacting gene with SEPT9. Immunostaining of CD206 in cervical cancer and polarization of macrophages (M2) were evaluated by immunofluorescence and WB. The Cancer Genome Atlas (TCGA) database was used for screening the potential miRNAs induced by SEPT9.

**Results:**

Hyper-methylation of SEPT9 detects cervical cancer and normal tissues, normal+CIN1 and CIN2+CIN3+cancer with high sensitivity and specificity (AUC = 0.854 and 0.797, respectively, *P* < 0.001). The mRNA and protein expression of SEPT9 was upregulated in cervical cancer tissues when compared to para-carcinoma tissues. SEPT9 promotes proliferation, invasion, migration, and influences the cell cycle of cervical cancer. SEPT9 interacted with HMGB1-RB axis increases irradiation resistance. Furthermore, SEPT9 mediated miR-375 via the tumor-associated macrophages (TAMs) polarization, affecting the resistance to radiotherapy in cervical cancer.

**Conclusions:**

These findings give us the evidence that SEPT9 methylation could be a biomarker for cervical cancer diagnoses. It promotes tumorigenesis and radioresistance of cervical cancer by targeting HMGB1-RB axis and causes polarization of macrophages by mediating miR-375. We suggest SEPT9 could be a potential screening and therapeutic biomarker for cervical cancer.

**Electronic supplementary material:**

The online version of this article (10.1186/s13148-019-0719-9) contains supplementary material, which is available to authorized users.

## Background

Cervical cancer is the fourth most common cancer in female and the fourth leading cause of cancer-related deaths [1]. According to the latest global data released by the International Agency for Research on Cancer, there were 569,847 new cases of cervical cancer worldwide in 2018, and 311,365 deaths attributed to cervical cancer [2]. Although HPV vaccination and early screening programs have emerged as effective strategies in disease prevention [3], the high incidence rates are observed in low- and middle-income countries (LMICs) [4]. And even though there has been a marked increase in survival rate from cervical cancer in China, this increase was still lower than some developed countries such as Canada, Norway, and Australia [5, 6].

At present, DNA methylation-based assays that can be used as a biomarker for the detection of advanced CIN2/3 lesions [7] and a lot of methylated genes have been described as promising markers for the management of HPV-positive women, such as SOX1, PAX1, JAM3, EPB41L3, CADM1, and MAL [8–10]. It is significant to explore and develop more sensitive and specific DNA methylation assays in order to further improve the early detection of cervical cancer.

According to the NCCN guidelines, the standard treatment of advanced cervical cancer is a combination of γ-irradiation and cisplatin-based chemotherapy [11]. However, when radiotherapy and chemotherapy are given concurrently, both acute and late toxicity become important concerns [12]. An epidemiologic and clinical analysis of cervical cancer from Japan showed poorer prognosis for younger age patients in the radiation-based treatment group despite a rise in relative survival rates shown by the recent statistics; hence, pointing towards the requirement of further improvement in therapeutic modalities for more advanced cases with distant metastasis [13]. Although tumor size and FIGO stage may efficiently serve as markers for responsiveness to radiotherapy, the resistance of tumor cells to radiation remains a major therapeutic hurdle. Therefore, it is necessary to find new molecular targets to investigate the exact mechanism of cervical cancer and assess the radiation sensitivity.

Septins constitute a family of GTP-binding proteins that are involved in a variety of biological processes such as cytoskeleton formation, cell division and tumorigenesis [14]. Promoter hyper-methylation of SEPT9 has been confirmed as a potent biomarker in colorectal cancer [15] and some other cancers. SEPT9 has been identified as a potential oncogene, and its overexpression was observed in several carcinomas, including breast [16], ovarian [17], head and neck [18], and prostate [19]. However, the mechanism of SEPT9 in cervical cancer still remains largely unexplored.

In the present study, we firstly found that hyper-methylation of SEPT9 exhibits a high sensitivity and specificity in the diagnosis of cervical cancer. Up- and downregulation of SEPT9 affected the biological behavior of cervical cancer cells through the regulation of HMGB1. Moreover, we found that SEPT9 mediated miR-375 could enhance the cell resistance to ionizing radiation via affecting the tumor-associated macrophages (TAMs) polarization.

## Methods

### Tissue specimens

A total of 80 cases of specimens, including paired cervical cancer tissues and para-carcinoma normal tissues, were collected from cervical cancer patients who underwent surgery in the Qilu Hospital of Shandong University from 2013 to 2017. The patients were diagnosed with cervical cancer on pathological basis. All experiments in this study were approved by the Ethics Committee of the Qilu Hospital of Shandong University. The patients and their families were informed of specimen collection, and the informed consent forms were signed.

### DNA extraction, bisulfite modification, and methylation-specific PCR

Genomic DNA from tissues was extracted using a QIAamp DNA Mini Kit (Qiagen, Germany). Genomic DNA extracted from tissues was treated with sodium bisulfite using an EZ DNA Methylation-GoldTM kit (Zymo Research, USA). Multiplex methylation-specific PCR (MSP) was performed in a 25-μL volume reaction system that consisted of 50 ng sodium bisulfite-treated DNA, isometric mixture of gene primers 3 μL, 2×Master Mix 12.5 μL (Qiagen, Germany) and ddH_2_O. The multiplex MSP primer sequences for SEPT9 [20] and β-actin [21] are illustrated in Additional file 2: Table S2. We performed Hot-start PCR at an annealing temperature of 60 °C using hot-start Taq DNA polymerase (Thermo Fisher Scientific).

### Cell culture conditions

The cervical cancer cell lines HeLa and CaSki were purchased from the American Type Culture Collection (ATCC, Rockville, MD, USA) and cultured in DMEM and RPMI-1640 (HyClone Laboratories, Inc., Logan, UT, USA) supplemented with 10% fetal bovine serum at 37 °C in an incubator with 5% CO_2_.

### Real-time PCR

Total RNA was isolated from tissues and cells using the TRIzol reagent (Invitrogen, USA). Purified RNA was reversely transcribed into cDNA using a PrimerScript RT Reagent kit (TaKaRa, Japan). The amplification was performed using the SYBR Green dye by Roche real-time PCR instrument. The β-actin was used as an internal reference. The experiments were repeated in triplicate to confirm the findings.

### Immunohistochemistry

Histologically normal and cervical cancer samples were fixed in formalin and embedded in paraffin, and 4-μm thick tissue sections were cut. After blocking the endogenous peroxidase, antigen retrieval was performed followed by serum albumin blocking for the non-specific binding sites. The tissue sections were incubated at 4 °C for 12 h with mouse anti-SEPT9 antibodies. Incubation with secondary antibody was performed using HRP-labeled goat anti-mouse antibody for 30 min at room temperature, followed by treatment with DAB for 1 min. Staining scores were calculated using Image Pro Plus 6.0.

### Plasmid and transfections

Small interfering RNA (siRNA) to knockdown SEPT9, HMGB1, and negative control were purchased from GenePharma (Shanghai, China). Transient transfections were carried out using Lipofectamine 2000 (Invitrogen, USA) following the protocol from the manufacturer. To establish stable transfections, lentiviral transfer plasmid was purchased from Hanbio (Shanghai, China). The stably transfected cells were incubated in the medium containing puromycin, and the puromycin-resistant colonies were observed approximately 2 days after transfection.

### Cell viability assay, migration and invasiveness assays, and cell cycle analysis

CCK8 was used to assess cell viability. Cells (2 × 10^3^/well) were seeded into a 96-well plate and incubated overnight in the previously described conditions. Following this, the medium was removed, and the cells were washed twice with PBS. Medium (90 μL) and CCK8 (10 μL) were subsequently added to each well and incubated for 2 h at 37 °C; a microplate reader spectrophotometer was used to measure the optical density (OD) at 450 nm.

For migration assay, we used Boyden chambers (pore size, 8 μm) (BD Biosciences). The cells (5 × 10^4^ cells in 0.2 ml of medium) were placed in the upper chamber, and 0.70 ml of DMEM containing 15% FBS was placed in the lower chamber. After 24-h incubation, cells on the upper side of the filters were removed with cotton-tipped swabs, and the filters were fixed in methanol for 10 min and stained with 0.05% crystal violet. Cells underneath the filters were observed and counted under a microscope. For the invasion assay, same conditions were applied as were described for migration assay except that the invasion chambers were coated with biocoat Matrigel.

Cell cycle analysis was performed by flow cytometry. Harvested cells were fixed with 90% ethanol at − 20 °C for 1 h. The cells were washed twice with cold PBS (1×), suspended in 500 μL PBS, and incubated with 20 μL RNase at 37 °C for 30 min. Then, the cells were stained with 400 μL propidium iodide on ice for 30 min and filtered with a 53-μm nylon mesh. Cell cycle distribution was calculated from 10,000 cells with ModFit LT software using Beckman Coulter (Beckman, USA).

### Immunoprecipitation and Western blot

Subconfluent proliferating cells in 100-cm^2^ dishes were harvested, collected in lysis buffer and left on ice for 30 min, sonicated, and centrifuged at 15000 rpm for 15 min at 4 °C. Supernatants were collected. Each immunoprecipitation (IP) was carried out using 5-μg antibody and 500-μg protein. The precipitated proteins were collected using protein A+G beads, washed, eluted in boiling Laemmli sample buffer, and subjected to Western blotting. Briefly, 100-μg protein from each group was fractionated on 10% SDS-polyacrylamide gels and transferred to nitrocellulose membranes (Millipore, Bedford, MA, USA). The membranes were then blotted with primary antibodies followed by the secondary antibody and developed with enhanced chemiluminescence reagent (Invitrogen, USA). The primary antibodies used in this study were obtained from Abnova (Beijing, China): SEPT9(H00010801-M01) and Cell Signaling Technology (Beverly, MA, USA): β-actin (4970), p-mTOR (5536), p-Akt (4060), CyclinD1 (2978), CyclinA2 (91500), E-cadherin (3195), Snail (3879), RB (9309); Abcam (Cambridge, MA, USA): HMGB1 (ab79823), E2F1 (ab218527), P16 (ab108349), CD206 (ab64693), Arg1 (ab133543), and INOS(ab178945).

### X-irradiation

X-irradiation experiments were performed at the Irradiation Facility of Shandong University. The medium was replaced prior to irradiation. Cells were divided into different groups and exposed under (0, 2, 4, 6, 8, 10 Gy) X-rays separately with a Siemens Primus Accelerator machine.

### Immunofluorescence

The cell lines were plated onto cover-slips and treated, respectively. After 48 h, cells were fixed with 4% paraformaldehyde for 10 min at room temperature. All slides were then washed with PBS and blocked with Goat serum at room temperature for 30 min. Cells were incubated with primary antibodies (γH2AX: Abcam, ab2893, 1:400; CD206: Abcam, ab64693, 1:400) at 4 °C overnight, washed with PBS, and then incubated with appropriated secondary antibodies for 1 h at room temperature. Cells were examined immediately using Nikon C-HGFI Intensilight Fiber Illuminator (Nikon, Japan) fluorescence microscope.

### In vivo studies

Animal studies were performed according to institutional guidelines. HeLa cells were stably transfected with NC or SEPT9 overexpression vectors. A total of 5 × 10^6^ viable cells were injected into the right flanks of nude mice. Tumor sizes were measured using a Vernier caliper every 5 days, and the tumor volume was calculated using the following formula: volume = 1/2 × length × width^2^. At 30 days after implantation, the mice were exposed to X-ray irradiation (5 Gy every 3 days, total 15 Gy). Then after 12 days, they were sacrificed, the tumors were dissected, and tumor weights were measured.

### Statistical analysis

The results were expressed as mean ± SEM. Statistical analyses were performed using SPSS version 18.0 software. Statistical comparisons between 2 samples were performed using the Student’s *t* test, and those variances between 3 or more groups were analyzed by one-way ANOVA test. The correlation between the two gene expressions was analyzed by Spearman’s rank correlation. The sensitivity, specificity, and the area under the ROC curve (AUC) were calculated for diagnostic evaluation (normal vs. cancer, normal+CIN1 vs. CIN2+CIN3+cancer). Youden index (sensitivity+specificity-1) was used to calculate the optimal cut-off value, the point on the ROC curve with the shortest distance value from the top left corner (point: 0,1). Differences were considered significant when *P* < 0.05.

## Results

### SEPT9 methylation assay detects cervical cancer with high sensitivity and specificity

**Fig. 1:**
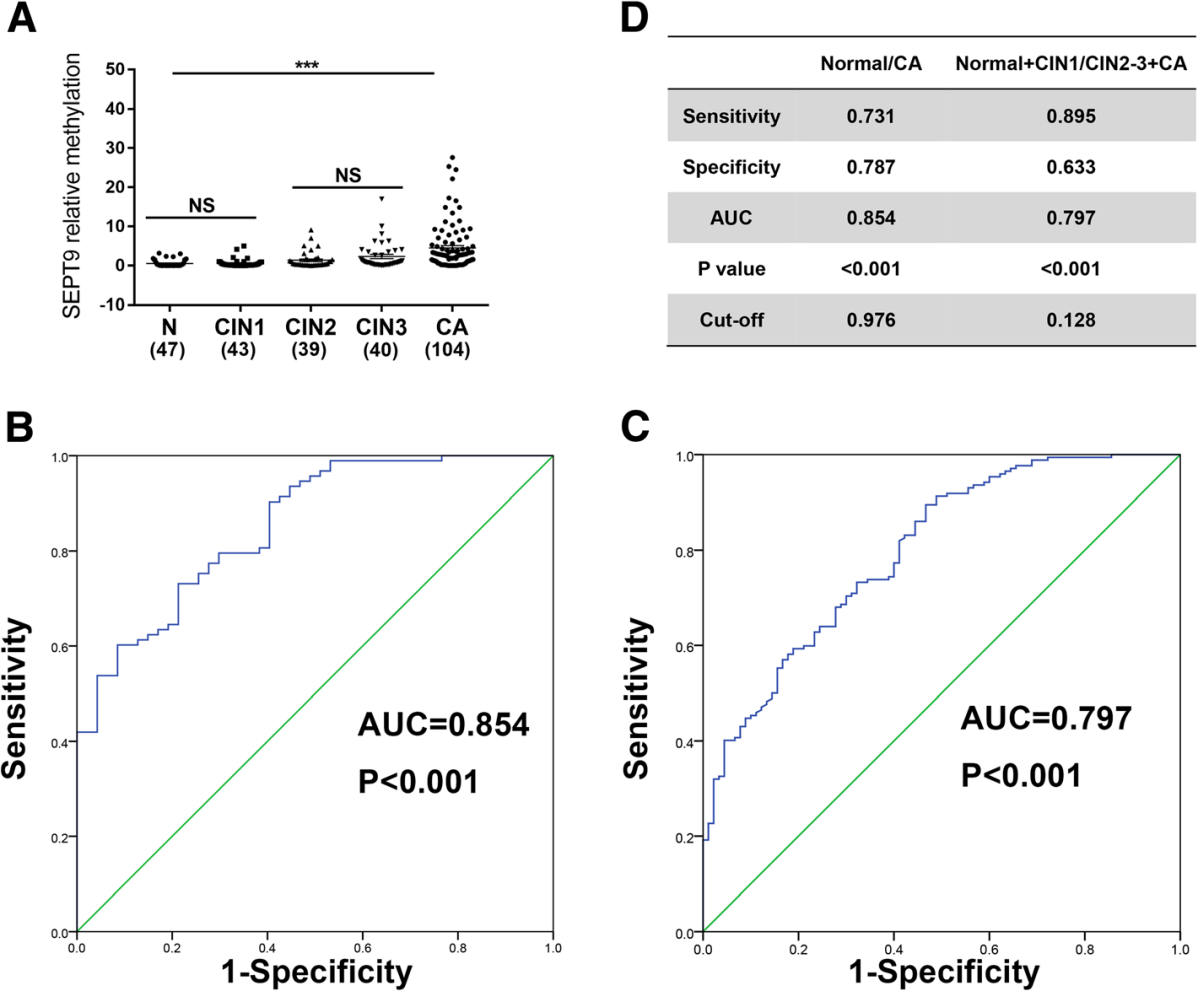
SEPT9 methylation assay detects cervical cancer with high sensitivity and specificity. **a** Methylation of SEPT9 in normal tissues, CIN1, CIN 2, CIN 3, and cervical cancer tissues. **b**, **c** Receiver operating characteristic (ROC) curve analysis of normal with cancer tissues and normal+ CIN 1 with cancer+ CIN 2+CIN 3 tissues. The area under the ROC curve (AUC) was calculated for the diagnosis of cervical cancer tumors. **d** Comparison in the two sets on the bases of sensitivity, specificity, AUC, cut-off, and *P* value

The normal samples, CIN 3 samples, and tumor samples (10 cases/group) were analyzed using the Illumina Methylation EPIC 850k Beadchip. RnBeads package was used for statistical analyses [22], SEPT9 displayed a significant hyper-methylation (tenth) (Additional file 1: Table S1). We detected aberrant methylation of SEPT9 in normal tissues (*n* = 47), CIN 1 (*n* = 43), CIN 2 (*n* = 39), CIN 3 (*n* = 40), and cervical cancer tissues (*n* = 104) by MSP. Our results revealed that SEPT9 was highly methylated in cervical cancer tissues as compared to the normal tissues (Fig. 1a). Based on this, we estimated the diagnostic significance of methylated SEPT9 in cervical cancer. ROC curve indicated a high diagnostic value of methylated SEPT9 in cervical cancer tissues compared to the normal tissues, with an area under the curve (AUC) of 0.854, which corresponds to a sensitivity of 0.731 and specificity of 0.787 (Fig. 1b). Meanwhile, when we compared normal+CIN1 group to CIN2+CIN3+cancer group, ROC analysis revealed an AUC of 0.797 for cancer with a sensitivity of 0.895 and specificity of 0.633 (Fig. 1c).

### SEPT9 is overexpressed in cervical squamous cell carcinoma (CSCC)

**Fig. 2:**
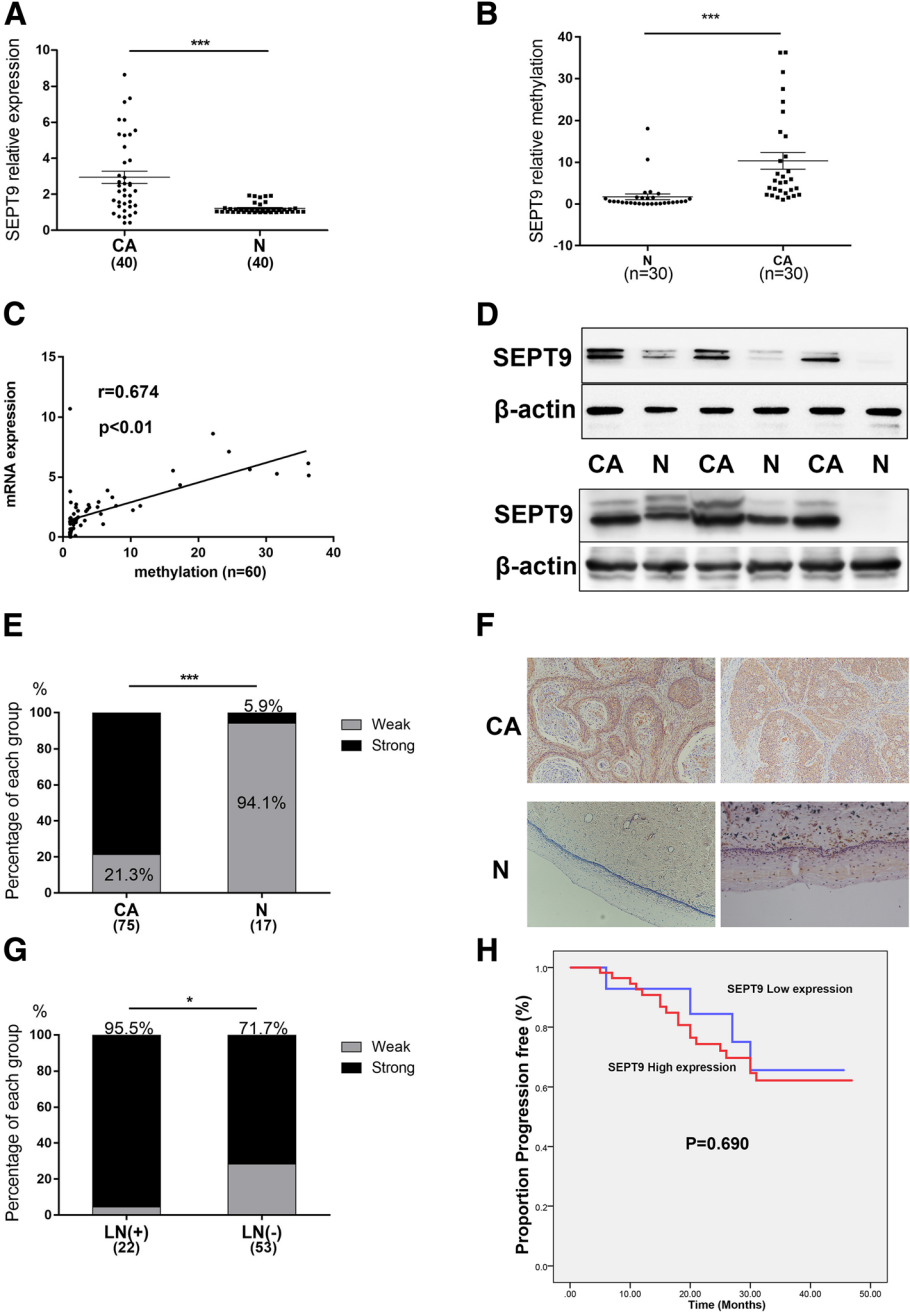
SEPT9 is overexpressed in cervical squamous cell carcinoma (CSCC). **a** SEPT9 mRNA expression in cervical cancer samples and adjacent normal cervical samples. **b** Methylation of SEPT9 in cervical cancer tissues and para-carcinoma tissues. **c** The positive correlation between SEPT9 DNA methylation and mRNA expression. **d** The protein expression levels of SEPT9 in the cervical cancer tissues and normal para-carcinoma tissues were measured by Western blot. **e** The distribution of immunohistochemical score, showed in histogram, indicates that SEPT9 has a significantly higher expression in CSCC. **f** Representative images of immunohistochemical staining of SEPT9 in the cancer tissues and normal cervical tissues (× 100 magnification). **g** The high expression of SEPT9 accounted for a higher proportion in the patients with positive lymph nodes. **h** A Kaplan-Meier depiction of progression-free survival by the expression of SEPT9. *P < 0.05, ***P < 0.001

The mRNA expression of SEPT9 was found to be significantly higher in CSCC (*n* = 40) samples compared with para-carcinoma (*n* = 40) samples (*P* < 0.001) (Fig. 2a). We examined the levels of SEPT9 methylation in 30 pairs of cervical cancer and para-carcinoma tissues. As shown in Fig. 2b, we found that SEPT9 was higher methylation in cancer tissues than in the paired para-carcinoma tissues (*P* < 0.001). We found there was a positive correlation which existed between SEPT9 DNA methylation and mRNA expression from the cervical cancer tissues and paired para-carcinoma tissues (*r* = 0.674, *P* < 0.01; Fig. 2c).

At the protein level as well, the expression of SEPT9 was higher in CSCC (*n* = 20) samples in comparison to the para-carcinoma tissue (*n* = 20) samples (Fig. 2d). To further confirm the role of SEPT9 in CSCC, immunohistochemical staining was performed to compare its expression level in the CSCC (*n* = 64) and normal cervical epithelial samples (*n* = 17). It was found that SEPT9 had significantly higher intensity of immunostaining in CSCC than in normal cervical epithelium (78.7% vs 5.9%) (Fig. 2e, f). Analyzing the clinical data of the patients (Table 1), we found that the overexpression of SEPT9 accounted for a higher proportion in the patients with positive lymph nodes (Fig. 2g). Figure 2h is a Kaplan-Meier depiction of progression-free survival by the expression of SEPT9. There was no statistical difference between SEPT9 high-expression group and low expression group (*P* = 0.690).

**Table 1:** Association between SEPT9 expression and the clinicopathological features of patients with cervical cancer

		SEPT9 expression		
Characteristics	Patient	Low	High	*P*
	*n* = 75	*n* = 16	*n* = 59	
Age				
< 50	49	13	36	0.131
≥ 50	26	3	23	
FIGO stage				
I	50	10	40	0.69
II	25	6	19	
Tumor diameter				
≤ 4 cm	57	12	45	0.916
> 4 cm	18	4	14	
Differentiation grade				
High	5	1	4	0.94
Middle+low	70	15	55	
Lymph node metastasis				
Positive	22	1	21	0.022*
Negative	53	15	38	
Vasoinvasion				
Yes	20	3	17	0.419
No	55	13	42	

*Significant *P* values (*P* < 0.05)

### SEPT9 promotes proliferation/invasion/migration and influences cell cycle of cervical cancer cells in vitro

**Fig. 3:**
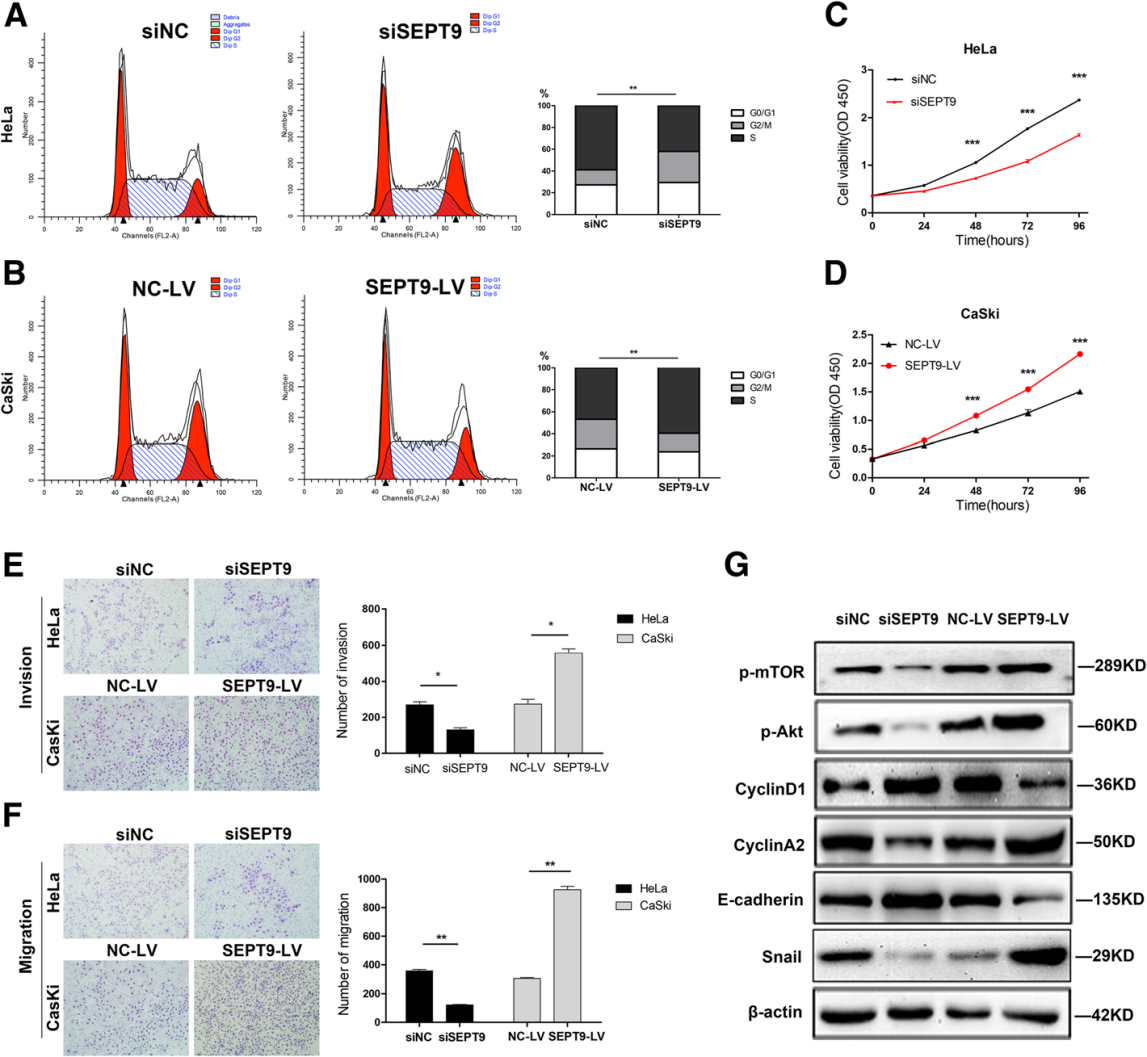
SEPT9 affects the biological behavior of cervical cancer cells. **a**, **b** Transfected siNC and siSEPT9 in HeLa and transfected NC-LV and SEPT9-LV in CaSki, cell cycle analysis by flow cytometry. **c**, **d** Cell viability of Hela and CaSki cells was measured by the CCK8 assay. **e**, **f** SEPT9 effects on the invasion and migration of cervical cancer cells with SEPT9 knockdown or overexpression. **g** Western blot analysis of p-mTOR, p-AKT, cylcinD1, cyclinA2, e-cadherin, and snail in SEPT9 knockdown and overexpression HeLa cells compared to control cells. **P < 0.01, ***P < 0.001. siNC, small interfering RNA negative control; siSEPT9, small interfering RNA targeting SEPT9; NC-LV, pHBLV-CMV-MCS-3flag-EF1-Zsgreen-puro negative control; SEPT9-LV, pHBLV-CMV-MCS-3flag-EF1-Zsgreen-puro tagged SEPT9

SEPT9 was down- or upregulated in HeLa and CaSki cell lines via transfection with siSEPT9 for knockdown and SEPT9-LV for overexpression. The data showed that the suppression of SEPT9 in HeLa and CaSki cells significantly decreased the cell proliferation. On the other hand, cell proliferation was increased after the upregulation of SEPT9 in both cell lines (Fig. 3c, d). Moreover, the cell cycle assay showed that the number of cells in G0/G1 phase was increased with suppression of SEPT9 in HeLa cells (27.29% vs 29.45%), while it was reduced in the G2/M phase (58.94% vs 42.01%) and vice versa (Fig. 3a, b). The results ascertained from transwell assay are shown in Fig. 3e and f. Cell migration and invasion ability were found to have significantly increased by SEPT9 upregulation. The proteins related to proliferation, EMT, and cyclin were also analyzed by Western blot. The results showed that the levels of p-mTOR, p-Akt, Snail, and cyclinA2 proteins were reduced by SEPT9 knockdown in CaSki cells, whereas increased by SEPT9 upregulation. The levels of E-cadherin and cyclinD1 proteins were obviously increased by SEPT9 downregulation and decreased when SEPT9 was upregulated (Fig. 3g).

### SEPT9 increases irradiation resistance in vitro and in vivo

**Fig. 4:**
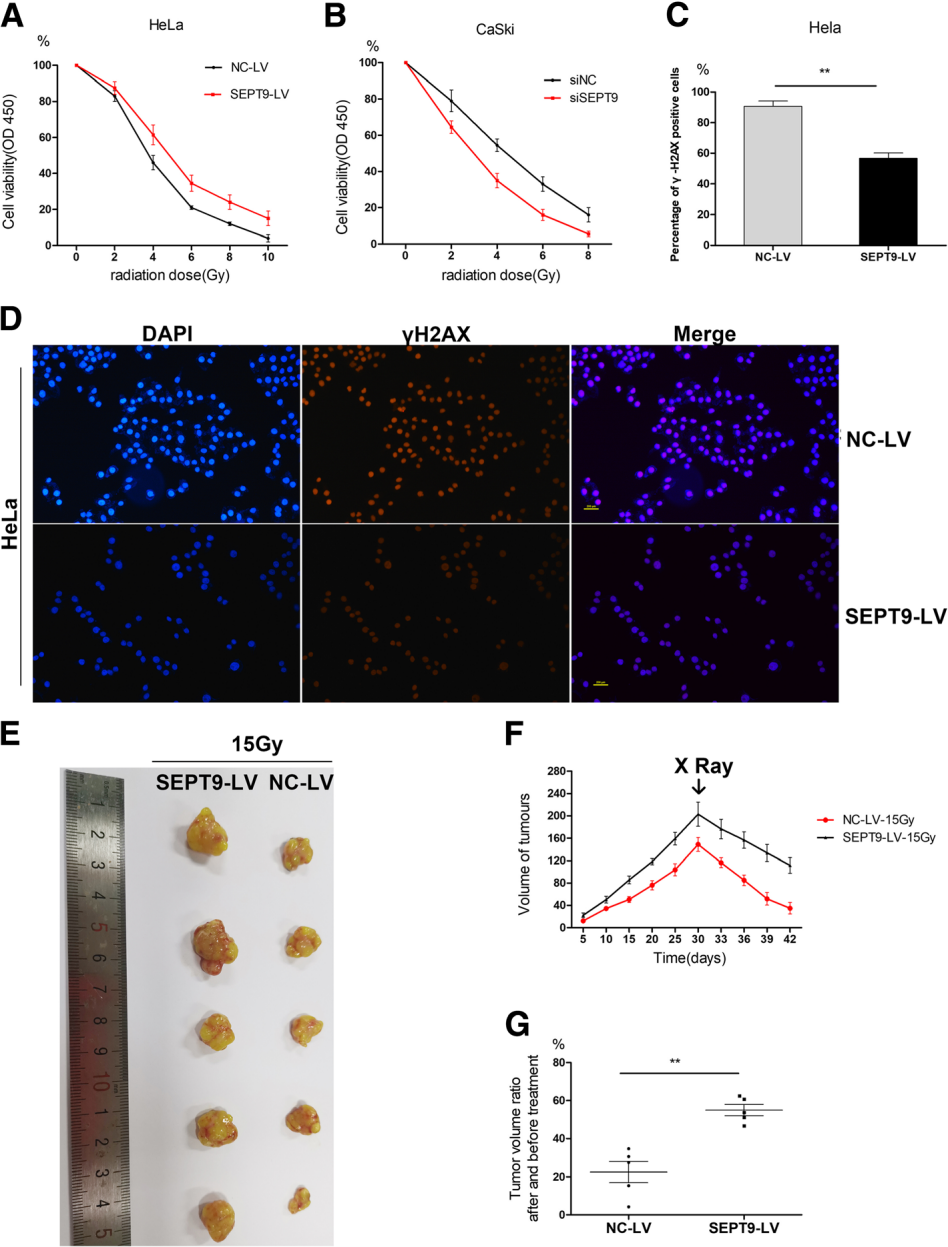
**a**–**g** SEPT9 increases irradiation resistance in *vitro* and in *vivo*. **a**, **b** Cell viability after different doses of irradiation treatment was increased by HeLa transfected siSEPT9 and decreased by CaSki transfected SEPT9-LV. **c**, **d** γH2AX expression and subcellular localization were detected using immunofluorescence in HeLa cells transfected with SEPT9 overexpression. **e** HeLa cells with or without SEPT9 overexpression were subcutaneously inoculated into nude mice. Each group contained 5 mice. **f** Growth curves of tumors in control/SEPT9 overexpression group and after 30 days the mice were given irradiation total 15Gy. **g** The tumor ratio after and before radiation treatment

The CCK8 assay showed higher sensitivity to irradiation in HeLa and CaSki cells after they were transfected with siSEPT9 and increased irradiation resistance after SEPT9 was upregulated (Fig. 4a, b). After SEPT9 was overexpressed, the number of γH2AX accumulation in cells was less than that in the negative control group after the same intensity of radiotherapy (Fig. 4c, d).

The subcutaneous tumor-bearing model of cervical cancer was established in nude mice, and the effect of SEPT9 on the proliferation of cervical cancer cells in animals was further verified. The results showed that the subcutaneous tumor volume in the SEPT9-LV group grew obviously larger compared to the control group in 30 days, but after irradiation, the tumor volumes all decreased (Fig. 4e, f). The average proportion of tumor size in SEPT9-LV group was much larger than that in the control groups after the same dose of radiation (Fig. 4g).

### SEPT9 interacts with HMGB1 and is negatively correlated with HMGB1

**Fig. 5:**
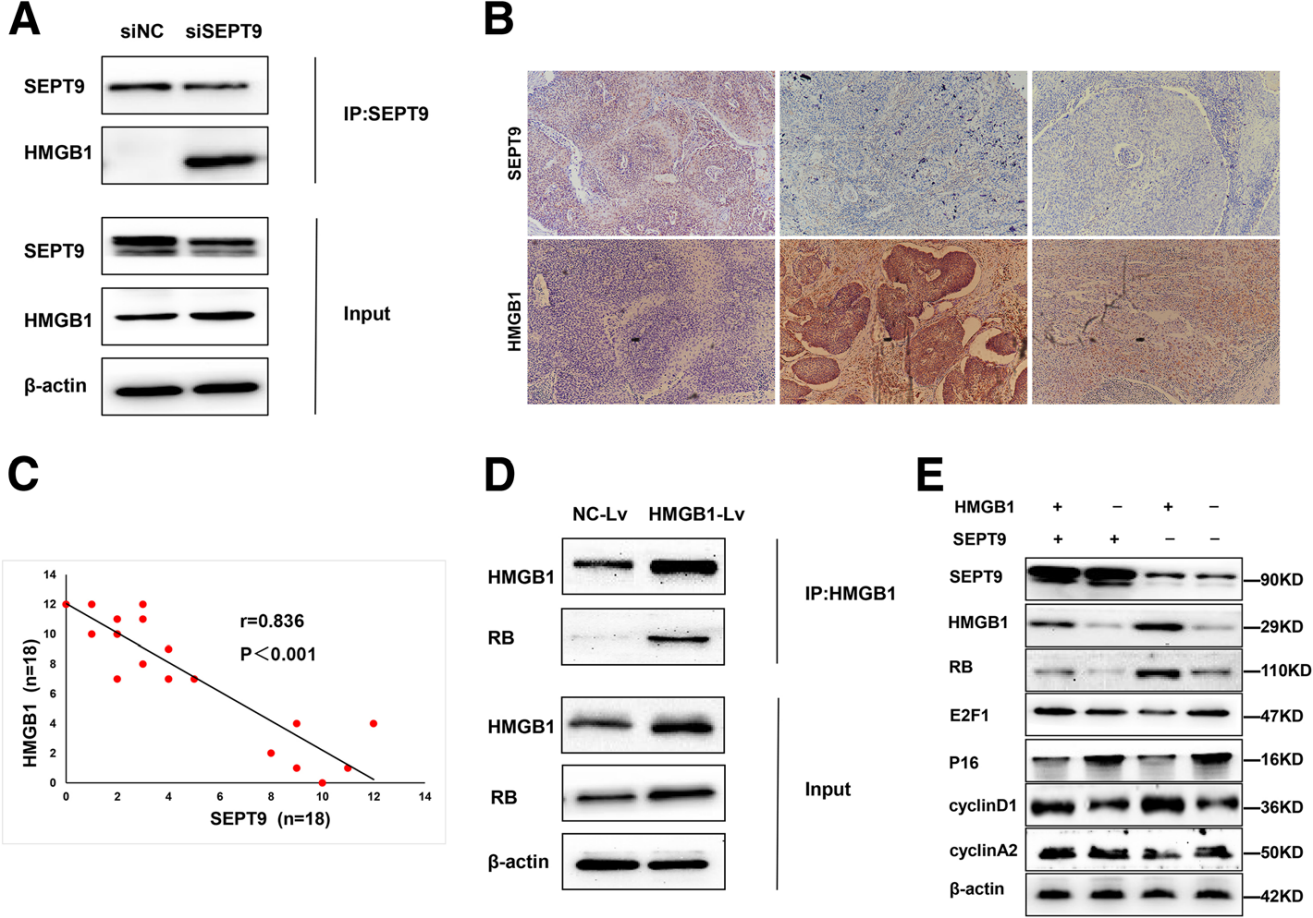
SEPT9 interacts with HMGB1 and enhances HMGB1-RB mediated transcription. **a**, **d** Nuclear extracts were prepared from HeLa and subjected to IP/Western blotting analyses. Endogenous association of SEPT9 and HMGB1, HMGB1, and RB. Representative results are shown from three independent experiments. **b** Representative images of immunohistochemical staining of HMGB1 in the cancer tissues that were stained by SEPT9. **c** The expression of SEPT9 is negatively correlated with HMGB1 expression. **e** SEPT9 regulates HMGB1 to affect RB, E2F1, p16, cyclin D1, and cyclin A2

According to the results from co-immunoprecipitation and liquid chromatography coupled with tandem mass spectrometry (LC-MS/MS), we found that HMGB1 protein interacted with SEPT9 (Additional file 3: Table S3 and Additional file 6: Figure S1). Thus, we assessed the interaction of SEPT9 protein with HMGB1 protein by immunoprecipitation (IP), the results from which showed that SEPT9 directly interacted with HMGB1 (Fig. 5a). Therefore, we examined HMGB1 expression in CSCC (Fig. 5b). Interestingly, it was found that the expression of SEPT9 was negatively correlated with HMGB1 (*r* = 0.836, *P <* 0.001; Fig. 5c). We also demonstrated suppression of HMGB1 significantly increased the cell proliferation and invasion but reduced the radiotherapy sensitivity, and vice versa. (Additional file 7: Figure S2)

We next verified that HMGB1 directly regulated RB (Fig. 5d). Then, we examined if SEPT9 imposed any effects on HMGB1-RB-mediated E2F transcription repression. As expected, exogenous SEPT9 supplementation was able to reverse the regulatory effect of HMGB1 on the expression of RB (Fig. 5e). The p16^CDKN2A^ protein prevents phosphorylation of the RB protein which restrains transcription factors E2F. As a result, the cyclin D1 and cyclin A2 were altered by the loss of p16.

### SEPT9 affects irradiation sensitivity by regulating macrophage polarization

**Fig. 6:**
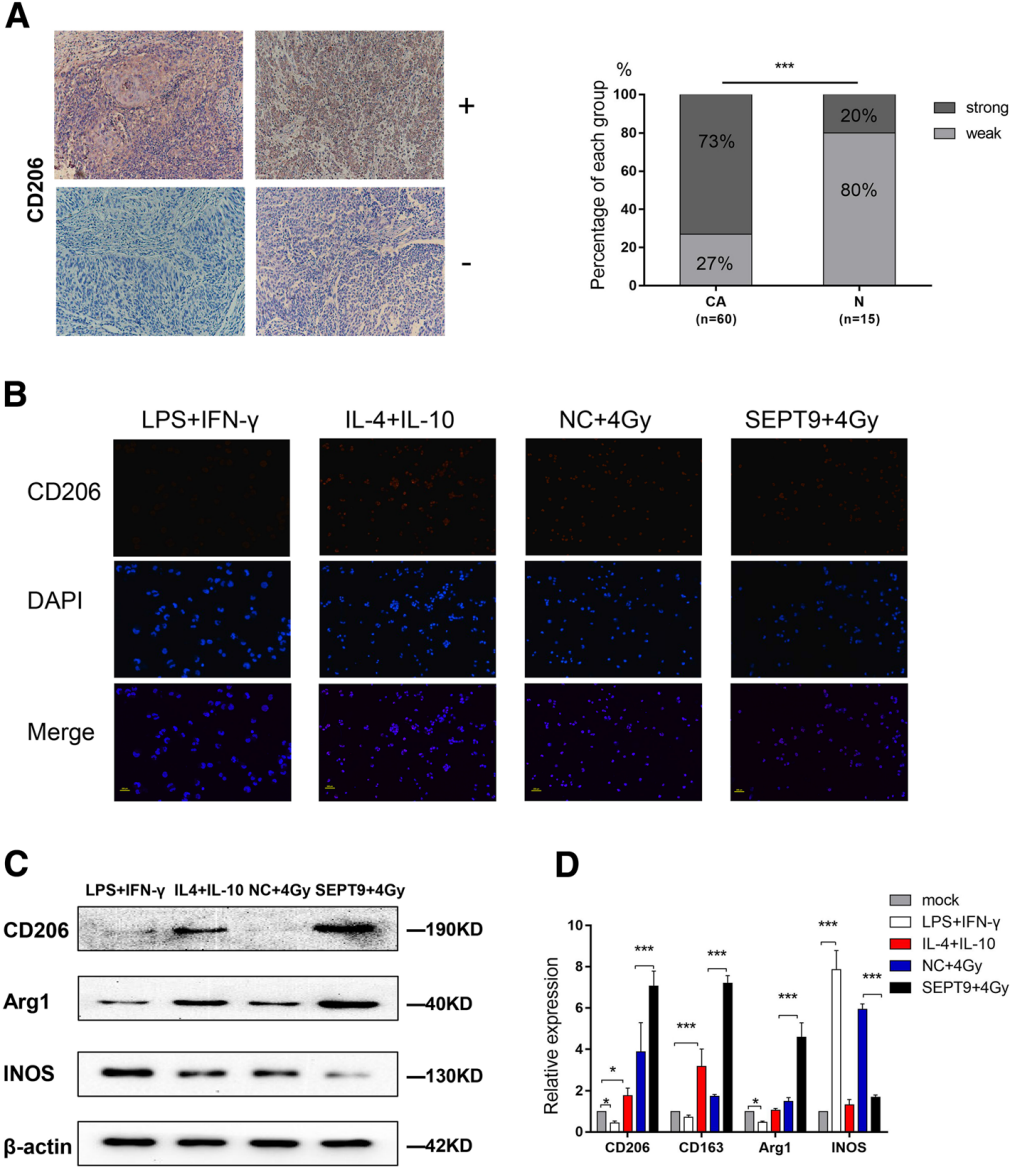
SEPT9 affects irradiation sensitivity by regulating macrophage polarization. **a** Representative images of immunohistochemical staining of CD206 in cervical cancer and in normal cervical epithelium. Forty-four of the 60 patients had CD206 positive in cancer tissues. **b** Immunofluorescence of CD206 after treatment of LPS (100 ng/ml)+IFN-γ (20 ng/ml), IL4 (20 ng/ml)+IL-10 (20 ng/ml), CM-NC-4Gy, CM-SEPT9-4Gy for 48 h. **c** Western blotting of CD206, Arg1, and INOS. **d** The relative mRNA expression of CD206, CD163, Arg1, and INOS by using qRT-PCR

CD206 had higher intensity of immunostaining in CSCC than in normal cervical epithelium (73% vs 20%) (Fig. 6a). After PMA treated for 24 h, THP-1 cells were treated with LPS+IFN-γ (negative control), IL-4+IL-10 (positive control) and conditioned medium that HeLa transfected with NC-LV or SEPT9-LV and 4-Gy irradiation (CM-NC-4Gy, CM-SEPT9-4Gy) for 48 h. Subsequently, the expression of CD206 by cells was examined via immunofluorescence. We found that fluorescence intensity was increased in CM-SEPT9-4Gy (Fig. 6b). We further analyzed the mRNA expression levels and protein levels of CD206, Arg1, and INOS. As shown in Fig. 6c and d, the expression of M2-phenotype marker CD206 and Arg1 were significantly increased in cells treated with CM-SEPT9-4Gy. In contrast, the same cell population showed reduced expression of M1-phenotype marker INOS.

### SEPT9 is mediated by miR-375 that transferred from tumor cells to TAMs

**Fig. 7:**
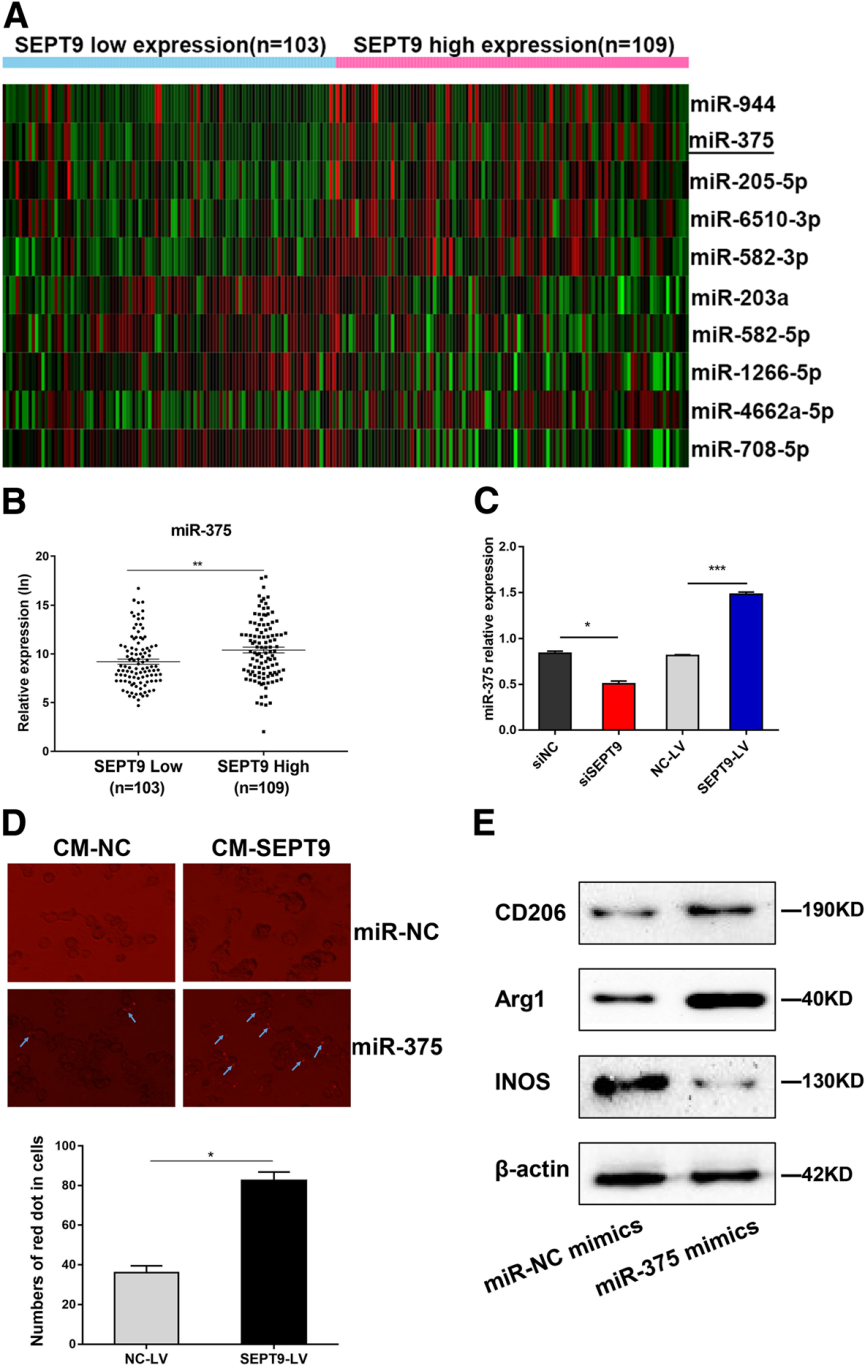
SEPT9 is mediated by miR-375 that transferred from tumor cells to TAMs. **a** Heat map diagram depicting expression of miRNAs dysregulated in CSCC from TCGA. **b** The expression of miR-375 in SEPT9 high and low expression patients from TCGA. **c** The relative miR-375 expression in CaSki transfected with siSEPT9 or SEPT9-LV. **d** CaSki transfected with SEPT9-LV or NC-LV were transiently transfected with RFP-labeled miR-375 or miR-NC were co-cultured with PMA-treated THP-1. Fluorescence microscopy was used to detect the red signals in THP-1 cells. **e** THP-1 cells were treated with PMA for 24 h and were transfected with miR-NC/375 mimics, then 20 ng/ml IL-4, and 20 ng/ml IL-10 were added for another 24 h. The proteins of both cells were isolated and the expression of M1 and M2 markers was detected

We identified SEPT9 related miRNA profile data from the Cancer Genome Atlas (TCGA) database, and heat map diagram depicted the expression of miRNAs dysregulated in cervical cancer tissues (Fig. 7a and Additional file 5: Table S5). The data from TCGA suggested that miR-375 had a higher expression level in SEPT9 high-expression patients (Fig. 7b and Additional file 4: Table S4). Similarly, the mRNA expression level of miR-375 was higher in CaSki cells transfected with SEPT9-LV, while low expression of miR-375 in transfected with siSEPT9 (Fig. 7c). Previous studies demonstrated that miR-375 could transfer from breast cancer cells to TAMs, so we further examined whether miR-375 could be transferred to modify the tumor microenvironment. As shown in Fig. 7d, we proved that miR-375 could be found more in TAMs that THP-1 treated with conditioned medium that HeLa transfected with SEPT9-LV (CM-SEPT9). Then, we explored the effect of miR-375 overexpression on TAMs phenotypes. THP-1 cells were treated with PMA for 24 h and transfected with miR-NC mimics and miR-375 mimics, then added IL-4 and IL-10 for 24 h for induction of M2 phenotype. The detection of protein levels showed M2 markers (CD206, Arg1) was significantly increased in miR-375 group while M1 markers INOS was remarkably decreased (Fig. 7e).

## Discussion

A large number of studies have shown that SEPT9 methylation testing could be a powerful diagnostic tool for many diseases, such as hepatocellular carcinoma [23], head and neck squamous cell carcinomas [24], invasive bladder cancer [25], and so on. One of the most remarkable breakthroughs, a sensitive blood-based colorectal cancer (CRC) screening test, uses the SEPT9 biomarker and specifically detects a majority of CRCs [26]. In April 2016, the plasma-based SEPT9 methylation assay, developed as the Epi proColon test, became the first blood test approved by the FDA for CRC screening. The US Preventive Services Task Force recommends screening for cervical cancer every 5 years with HPV testing in combination with cytological test in women [27]. In our study, the methylated SEPT9 test detected cervical cancer with 73.1% sensitivity and 78.7% specificity in tumor tissues. The efficiency and feasibility of the test conducted alone or in combination with conventional HPV DNA test and cytology in cervical cancer screening need to be further evaluated and verified.

A well-known mechanism operating in cancer has shown that CpG islands (CGIs) hyper-methylation at promoter sites represses transcription of genes acting as tumor suppressors [28]. Some studies have found an apparently positive correlation between methylation of gene body CGIs and gene expression [29, 30]. As an instance, we can consider a study conducted on a clinically relevant hepatocellular carcinoma mouse model that was used to verify that the expression levels of certain oncogene sets, characterized by hypermethylated CGIs either in their 5′-UTR or in the gene body, were found to be elevated in cancer [31]. Ball et al. [32] had confirmed that gene-body methylation in highly expressed genes is a consistent phenomenon throughout the human genome. Aran et al. [33] showed that positive correlation between gene-body hyper-methylation and gene expression is restricted to proliferative tissues and cell lines. Interestingly, our data showed that a clear positive correlation existed between SEPT9 DNA methylation and mRNA expression; however, the mechanism remains unclear. It has been reported that the knockdown of mammalian Septin family member, Septin 9 protein, isoform 1 (SEPT9_i1), a protein product of Septin 9 transcript variant 1(SEPT9_v1), affected cell proliferation, deregulated cell cycle, reduced angiogenesis, and decreased migration [34]. Our study systematically investigated the functions of SEPT9 in cervical cancer tumorgenesis both in vitro and in vivo. In this study, we found SEPT9 exhibited significantly differential expression between cervical cancer and para-carcinoma tissues. We also illustrated that SEPT9 could promote proliferation, invasion, and migration of cervical cancer cells and regulate the cell cycle.

Radiotherapy had been regarded as one of the most commonly used treatments for several cancers; however, several associated toxicities and evolution of insensitivity to radiotherapy have posed a big problem. In a study, BCL2 over-expressers showed significantly better response to salvage radiotherapy compared to low-expressers in prostate cancer [35]. Another study shows that NanoOlaparib could enhance the radio-sensitivity of radiation-resistant tumors lacking BRCA mutations [36]. Radiotherapy is the most effective therapy for advanced stages of cervical cancer. It is equally effective in treating early tumors as radical surgery, and it reduces local recurrences after surgery for patients with high-risk features [37, 38]. However, some cervical cancers develop resistance to radiotherapy, which can significantly compromise the clinical outcome. A study indicated ICAM-3 overexpression is associated with radiation resistance in cervical cancer cells [39]. The signaling from p-AKT could lead to radiation resistance in locally advanced cervical cancer [40]. Unfortunately, there is no available treatment specific to cervical cancer patients with radio-resistance. From this perspective, we investigate the relationship between SEPT9 and the radio-sensitivity in cervical cancer cells. We showed that the suppression of SEPT9 expression was partially responsible for the development of radio-sensitivity and that the ectopic expression of SEPT9 could be a potential strategy to develop radio-resistance in cervical cancer.

To examine the mechanism by which SEPT9 regulates radio-sensitivity in cervical cancer cells, we explored the association between SEPT9 and HMGB1, and were pleasantly surprised to find that SEPT9 was negatively correlated to HMGB1 in cervical cancer cell lines. HMGB1 is a member of the HMGB protein family, which has several biological functions inside as well as outside the cell [41]. Inside the cell, HMGB1 is an architectural chromatin-binding factor that binds DNA and promotes protein assembly on specific DNA targets [42]. Outside the cell, HMGB1 is a prototypical damage-associated molecular pattern, acting with cytokine, chemokine, and growth factor. In our study, a negative correlation between HMGB1 and SEPT9 expression was identified in cervical cancer by immunohistochemical staining. We not only established a direct interaction between SEPT9 and HMGB1 protein, but also confirmed the interaction between HMGB1 and RB, as it has been reported that intracellular HMGB1 functions as a tumor suppressor in breast cancer by directly binding to RB [43]. Interplay between HMGB1 and RB regulates Topoisomerase II alpha expression and subsequently, genomic stability [44].

It is well known that numerous macrophages are present in tumor tissues, termed tumor-associated macrophages (TAMs), directly affecting tumor progression in many cases [45]. TAMs can be designed as two functional types: M1 classically activated macrophages and M2 alternatively activated macrophages. Proinflammatory M1 macrophages are activated by IFN-γ and LPS, expressing INOS. M2 macrophages release immunosuppressive cytokines, expressing CD206 and Arg1, and secrete IL-10 and IL-4 [46, 47]. TAMs could contribute to tumor radiotherapy [48, 49]. Meng [47] has reported that depletion of TAMs by systemic or local injection of the macrophage-depleting liposomal clodronate before radiotherapy can increase the antitumor effects of ionizing radiation. Several studies have been demonstrated that miRNAs, mRNA, protein, long non-coding RNA, within exosomes, apoptotic bodies, or lipoprotein bound could induce the polarization of TAMs. A recent study showed miR-375, bound to low-density lipoproteins (LDL), transfer from apoptotic breast cancer cells to TAMs via the CD36 receptor to enhance macrophage migration and infiltration [50]. In our study, we also found that miR-375 could transfer from cervical cancer cell to TAMs (Fig. 7). We speculated that this transferation was through the same approach as in breast cancer cell line.

**Fig. 8:**
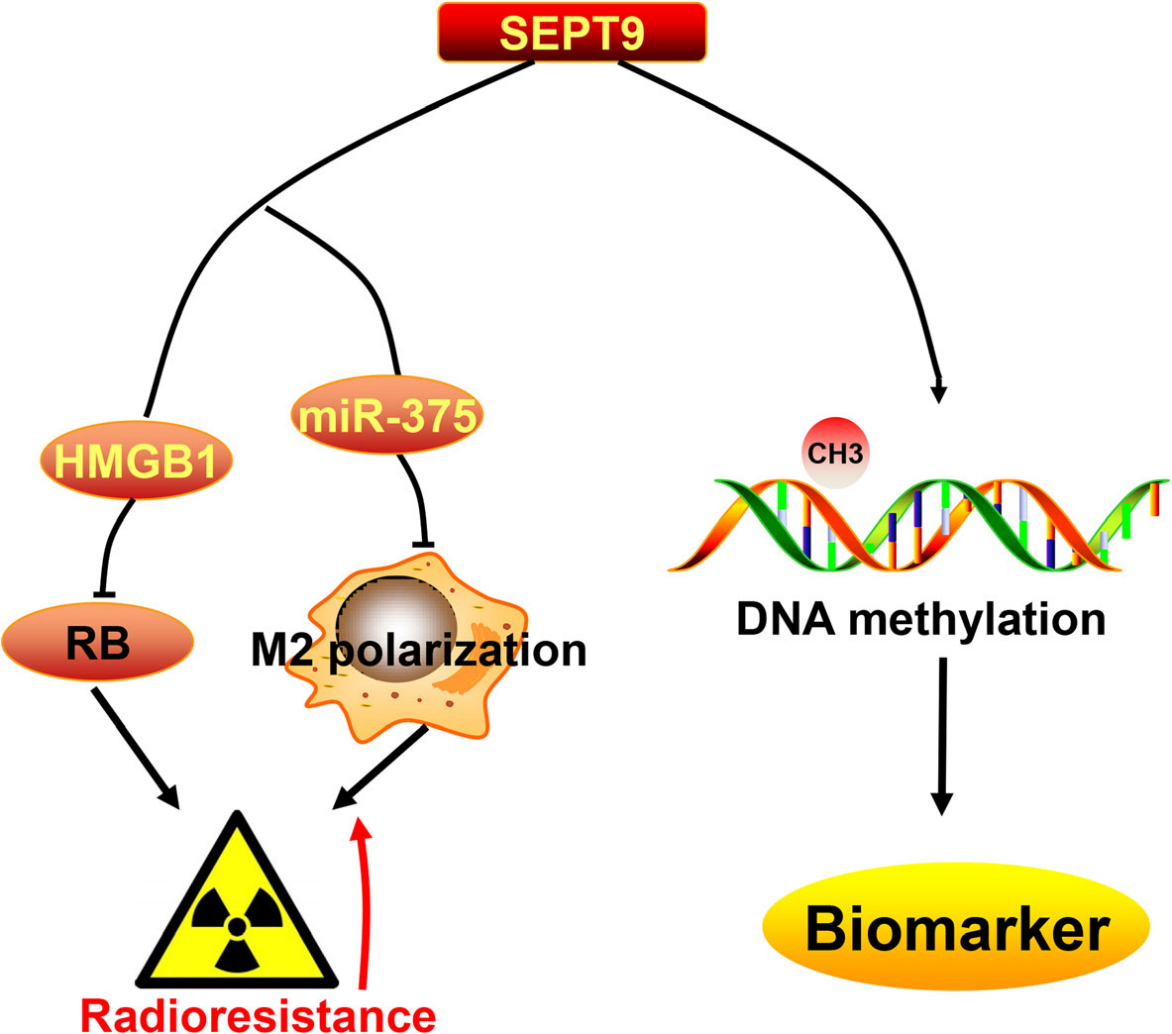
Pattern diagram of SEPT9 effect to cervical cancer

In summary, our study suggested that SEPT9 methylation has the potential to become an effective screening factor for cervical cancer prior to diagnosis. SEPT9 promotes tumorigenesis, cell migration, and invasion and regulates the sensitivity of radiotherapy by targeting HMGB1-RB pathway and regulating the polarization of macrophages via miR-375 (Fig. 8). Our study provided a possible screening indicator and radiotherapy sensitization target for cervical cancer.

## Conclusions

Hyper-methylation of SEPT9 could be a biomarker for cervical cancer diagnoses. But SEPT9 is overexpressed in cervical cancer tissues and acts the oncogenic role. SEPT9 could interact with HMGB1 and regulate the polarization of macrophages to increase irradiation resistance.

## Additional files

Additional file 1: **Table S1.** The detail information of SEPT9 CpG site in Illumina MethylationEPIC [850K] BeadArray. (DOCX 16 kb)

Additional file 2: **Table S2.** The primer sequence mentioned in the study. (DOCX 18 kb)

Additional file 3: **Table S3.** LC-MS/MS information about the proteins and corresponding peptides. (XLSX 20 kb)

Additional file 4: **Table S4.** SEPT9 related miRNA profile data from TCGA database (212 cases). (CSV 1240 kb)

Additional file 5: **Table S5.** The significant dysregulated miRNAs of SEPT9 related miRNA profile. (CSV 64 kb)

Additional file 6: **Figure S1.** Differential analyze the identified peptides between SEPT9-LV-CaSki and NC-LV-CaSki. (JPG 225 kb)

Additional file 7: **Figure S2.** HMGB1 affect the cell proliferation, invasion and the radiotherapy sensitivity. (A, B) Cell viability after transfected with siHMGB1 or HMGB1-LV. (C, D) SEPT9 effects on the invasion of HeLa and CaSki with HMGB1 knockdown or overexpression. (E, F) Cell viability after different doses of irradiation treatment were increased by HeLa transfected siHMGB1 and decreased by CaSki transfected HMGB1-LV. (JPG 2183 kb)

## Data Availability

The data used and/or analysis during the current study are available from the corresponding author on reasonable request.

## Abbreviations

LC-MS/MS: Liquid chromatography coupled with tandem mass spectrometry
CoIP: Co-immunoprecipitation
TAMs: Tumor-associated macrophages
MSP: Methylation-specific PCR
CSCC: Cervical squamous cell carcinoma
LPS: Lipopoly-saccharide
IFN-γ: Interferon-γ
IL-4: Interleukin 4
IL-10: Interleukin 10
PMA: Phorbol 12-myristate 13-acetate
TCGA: The Cancer Genome Atlas
CRC: Colorectal cancer
CGIs: CpG islands
LDL: Low-density lipoproteins
ROC: Receiver operating characteristic
