# Supplementary material for: Promoter methylation of SEPT9 as a potential biomarker for early detection of cervical cancer and its overexpression predicts radioresistance
Source: Clin Epigenetics. 2019 Aug 19;11:120. doi: 10.1186/s13148-019-0719-9 (PMC6700799; doi:10.1186/s13148-019-0719-9)
Supplement: Supplementary file 1 — Table S1. The detail information of SEPT9 CpG site in Illumina MethylationEPIC [850K] BeadArray. (DOCX 16 kb) [file 13148_2019_719_MOESM1_ESM.docx]

Supplementary Table 01. The detail information of SEPT9 CpG site in Illumina MethylationEPIC [850K] BeadArray

| **cgID** | cg20275528 |
| --- | --- |
| **Forward sequence** | TTCAGCTGAGCCAGGGGGCCTAGGGGCTCCTCCGGCGGCTAGCTCTGCACTGCAGGAGCG[CG]  GGCGCGGCGCCCCAGCCAGCGCGCAGGGCCCGGGCCCCGCCGGGGGCGCTTCCTCGCCGC |
| **UCSC refgene group** | 5'UTR;Body;5'UTR;Body;1stExon |
| **mean.CA/CIN3** | 0.349218664 |
| **mean.normal** | 0.031768275 |
| **mean.diff** | 0.317450388 |
| **mean.quot.log2** | 3.104382827 |
| **diffmeth.p. value** | 2.56E-07 |
