# Supplementary material for: Promoter methylation of SEPT9 as a potential biomarker for early detection of cervical cancer and its overexpression predicts radioresistance
Source: Clin Epigenetics. 2019 Aug 19;11:120. doi: 10.1186/s13148-019-0719-9 (PMC6700799; doi:10.1186/s13148-019-0719-9)
Supplement: Supplementary file 2 — Table S2. The primer sequence mentioned in the study. (DOCX 18 kb) [file 13148_2019_719_MOESM2_ESM.docx]

Supplementary Table 02. The primer sequence mentioned in the study

| **mSEPT9** | Forward primer | TTATTATGTCGGATTTCGCGGTTAAC |
| --- | --- | --- |
|  | Reverse primer | AAAATCCTCTCCAACACGTCCG |
| **mβ-actin** | Forward primer | TGGTGATGGAGGAGGTTTAGTAAGT |
|  | Reverse primer | AACCAATAAAACCTACTCCTCCCTTAA |
| **SEPT9** | Forward primer | ACCTTCTCATCAGGACGCAC |
|  | Reverse primer | GCTTCTGGCTCCTTCTCCTC |
| **β-actin** | Forward primer | AGTTGCGTTACACCCTTTCTTG |
|  | Reverse primer | CACCTTCACCGTTCCAGTTTT |
| **siSEPT9** | 5'-3' | GCACGATATTGAGGAGAAA |
| **siHMGB1** | 5'-3' | TCGGGAGGAGCATAAGAAGAA |
| **siNC** | 5'-3' | TTCTCCGAACGTGTCACGTAA |
