# Supplementary figures and images for: Promoter methylation of SEPT9 as a potential biomarker for early detection of cervical cancer and its overexpression predicts radioresistance
Source: Clin Epigenetics. 2019 Aug 19;11:120. doi: 10.1186/s13148-019-0719-9 (PMC6700799; doi:10.1186/s13148-019-0719-9)

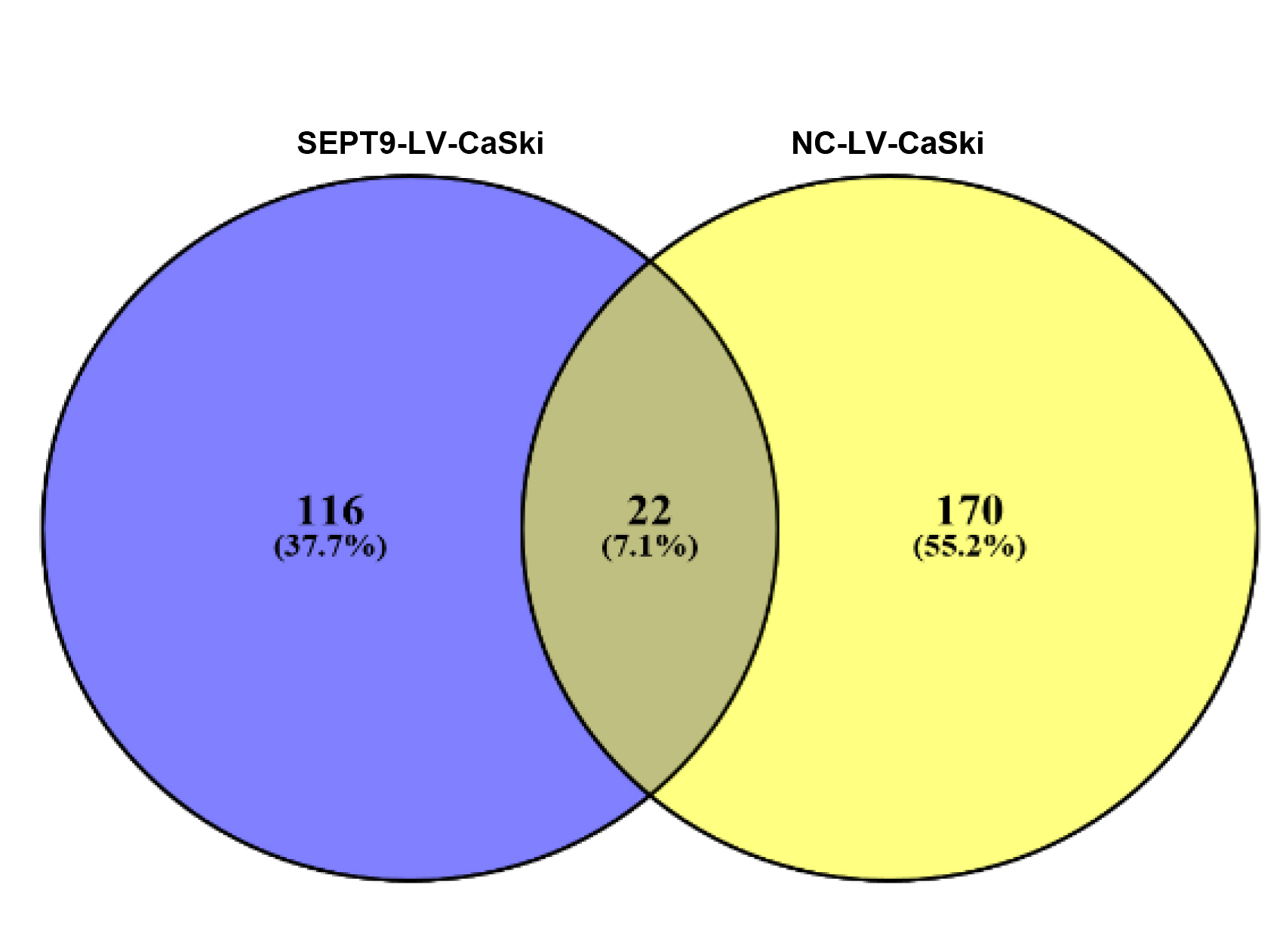

Supplement: Supplementary file 6 — Figure S1. Differential analyze the identified peptides between SEPT9-LV-CaSki and NC-LV-CaSki. (JPG 225 kb) [file 13148_2019_719_MOESM6_ESM.jpg]

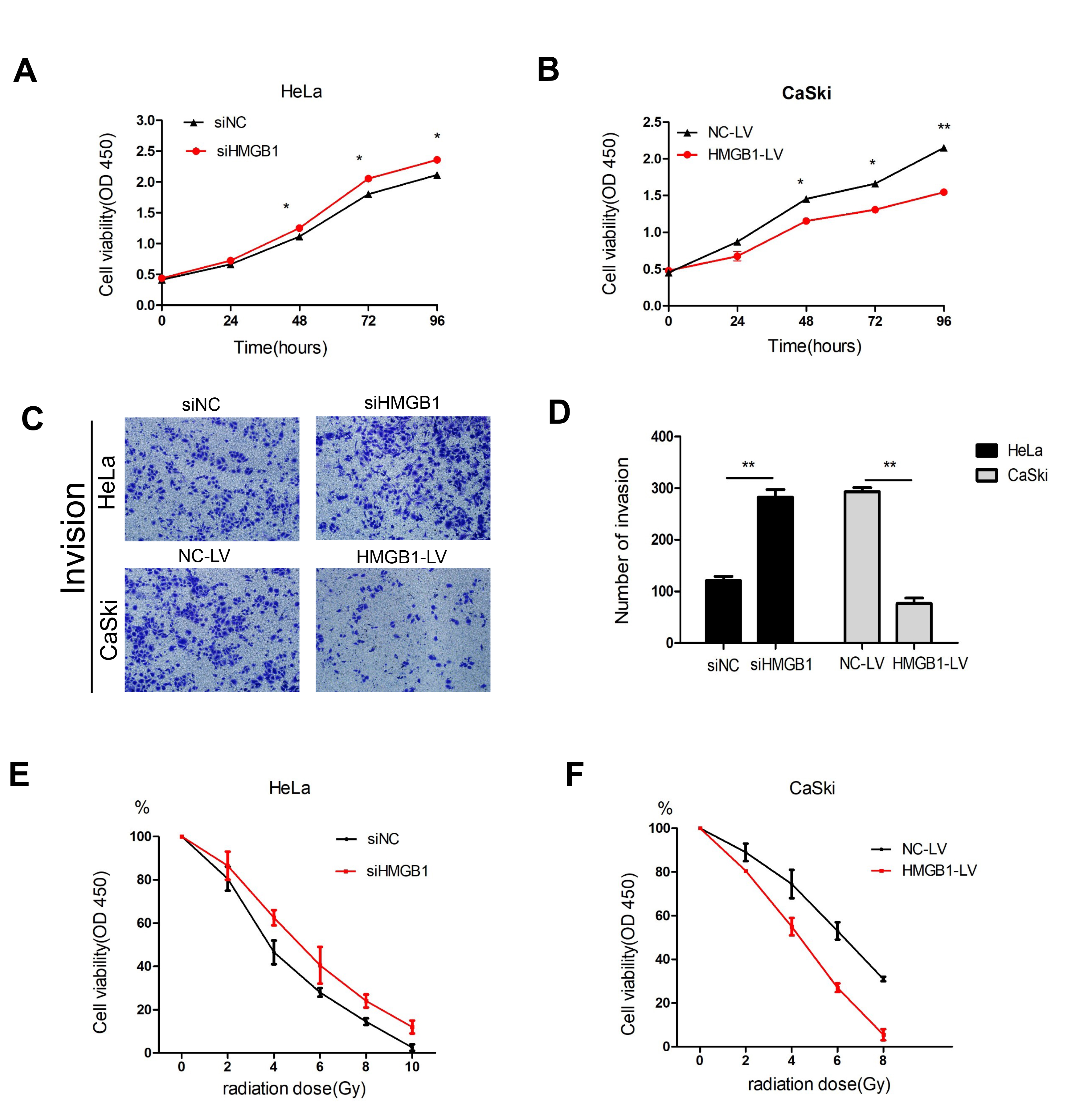

Supplement: Supplementary file 7 — Figure S2. HMGB1 affect the cell proliferation, invasion and the radiotherapy sensitivity. (A, B) Cell viability after transfected with siHMGB1 or HMGB1-LV. (C, D) SEPT9 effects on the invasion of HeLa and CaSki with HMGB1 knockdown or overexpression. (E, F) Cell viability after different doses of irradiation treatment were increased by HeLa transfected siHMGB1 and decreased by CaSki transfected HMGB1-LV. (JPG 2183 kb) [file 13148_2019_719_MOESM7_ESM.jpg]
